# The first RCT combining lifestyle with metformin to prevent cognitive impairement: experiences and updates from MET‐FINGER

**DOI:** 10.1002/alz70860_100196

**Published:** 2025-12-23

**Authors:** Alina Solomon, Mariagnese Barbera, Dinithi Perera, Jenni Lehtisalo, Celeste A de Jager, Geraint J Price, Francesca Mangialasche, Lefkos T Middleton, Tiia Ngandu, Miia Kivipelto

**Affiliations:** ^1^ Imperial College London, London, United Kingdom; ^2^ Karolinska Institutet, Stockholm, Sweden; ^3^ University of Eastern Finland, Kuopio, Finland; ^4^ FINGERS Brain Health Institute, Stockolm, Stockholm, Sweden; ^5^ Finnish Institute for Health and Welfare, Helsinki, Finland; ^6^ Karolinska University Hospital, Stockholm, Sweden; ^7^ FINGERS Brain Health Institute, Stockholm, Sweden; ^8^ The Ageing Epidemiology (AGE) Research Unit, School of Public Health, Imperial College London, London, England, United Kingdom; ^9^ The Ageing Epidemiology (AGE) Research Unit, School of Public Health, Imperial College London, London, United Kingdom, London, United Kingdom

## Abstract

**Background:**

Combining multidomain‐lifestyle interventions and pharmacological treatment could help address key challenges in dementia prevention. In this context, metformin is a promising candidate for drug‐repurposing, given the link between Type‐II Diabetes (T2D) and Alzheimer's Disease (AD). Emerging scientific evidence also suggests independent neuro‐protective effects.

MET‐FINGER is testing a FINGER 2.0 multimodal intervention, combining a structured multimodal lifestyle intervention with metformin, when appropriate (active arm), versus regular health‐advice (control/comparator arm), in an APOEε4‐enriched population of older adults at increased risk of dementia.

**Method:**

MET‐FINGER is a 2‐year international randomised, controlled, phase‐IIb proof‐of‐concept clinical trial, including metformin though a trial‐within‐trial design. 600 participants are being recruited at three sites (UK, Finland, Sweden). Participants randomly allocated to the FINGER 2.0 intervention who are at increased risk of T2D are further randomised to additionally receive metformin 2000mg/day, metformin 1000mg/day, or placebo (double‐blind). The lifestyle intervention is an optimised version of the FINGER model to be consistently delivered in three countries. Metformin is administered as Glucophage®XR/SR 500, (Merck KGaA, Darmstadt, Germany; 500mg oral tablets). Primary outcome is change in global cognition (combined z‐score of a neuropsychological test battery). Secondary outcomes are changes in individual cognitive domains; functional ability; and risk factors for AD/dementia. The feasibility, potential lifestyle‐metformin interaction, and disease‐modifying effects of the lifestyle‐metformin combination will be assessed as exploratory outcomes.

**Result:**

Recruitment started in January 2023. To date, 469 eligible participants have been identified and 336 have been randomised. The main challenges experienced have been linked to balancing the enrichment of APOEε4‐carriers in the study cohort and the eligibility rate for metformin treatment (e.g., APOEε4‐carriers were less likely to fulfil some eligibility criteria for metformin). The lessons learned, mostly pertaining to the recruitment process for future combination‐therapy studies, will be presented.

**Conclusion:**

MET‐FINGER is the first trial combining a multimodal lifestyle intervention with a putative disease‐modifying drug for the prevention of cognitive impairment. Its findings will provide crucial information for innovative precision‐prevention strategies aimed at cognitive impairment and dementia risk‐reduction, and evidence which will form the basis for a larger phase‐III trial design and other future research.

